# A Palindromic CpG-Containing Phosphodiester Oligodeoxynucleotide as a Mucosal Adjuvant Stimulates Plasmacytoid Dendritic Cell-Mediated T_H_1 Immunity

**DOI:** 10.1371/journal.pone.0088846

**Published:** 2014-02-24

**Authors:** Jun-ichi Maeyama, Hisakazu Takatsuka, Fumiko Suzuki, Ayumi Kubota, Satomi Horiguchi, Takako Komiya, Ichiroh Shimada, Eri Murata, Youko Osawa, Harukazu Kitagawa, Takasumi Matsuki, Masanori Isaka, Saburo Yamamoto, Sumiko Iho

**Affiliations:** 1 Department of Safety Research on Blood and Biological Products, National Institute of Infectious Diseases, Musashimurayama-shi, Tokyo, Japan; 2 Division of Legal Medicine, Niigata University Graduate School of Medicine and Dental Sciences, Niigata-shi, Niigata, Japan; 3 Department of Ophthalmology, Faculty of Medical Science, University of Fukui, Yoshida-gun, Fukui, Japan; 4 Laboratory of Host Defense, Faculty of Medical Sciences, University of Fukui, Yoshida-gun, Fukui, Japan; 5 Department of Anatomy and Neuroscience, Faculty of Medical Sciences, University of Fukui, Yoshida-gun, Fukui, Japan; 6 Department of Bacterial Pathogenesis and Infection, National Institute of Infectious Diseases, Musashimurayama-shi, Tokyo, Japan; 7 Forensic Medicine and Human Genetics, Faculty of Medical Sciences, University of Fukui, Yoshida-gun, Fukui, Japan; 8 Research and Education Program for Life Science and Translational Research Program, University of Fukui, Fukui-shi, Fukui, Japan; 9 Anesthesiology and Reanimatology, Faculty of Medical Sciences, University of Fukui, Yoshida-gun, Fukui, Japan; 10 Otorhinolaryngology-Head and Neck Surgery, Faculty of Medical Sciences, University of Fukui, Yoshida-gun, Fukui, Japan; 11 Chemical Substances Management, Administration Control Office, Emori & Co., Ltd., Fukui-shi, Fukui, Japan; 12 Department of Microbiology, Nagoya City University Medical School, Nagoya-shi, Aichi, Japan; 13 Central Laboratory, Japan BCG Laboratory, Kiyose-shi, Tokyo, Japan; Federal University of São Paulo, Brazil

## Abstract

**Background:**

CpG oligodeoxynucleotides (ODNs), resembling bacterial DNA, are currently tested in clinical trials as vaccine adjuvants. They have the nuclease-resistant phosphorothioate bond; the immune responses elicited differ according to the CpG ODN sequence and vaccination method. To develop a CpG ODN that can induce plasmacytoid dendritic cell (pDC)-mediated T_H_1 immunity through the mucosa, we constructed phosphodiester G9.1 comprising one palindromic CpG motif with unique polyguanosine-runs that allows degradation similar to naturally occurring bacterial DNA.

**Methods:**

T_H_1 and T_H_2 immunity activation was evaluated by cytokine production pattern and *T-bet/GATA-3* ratio in human peripheral blood mononuclear cells and mouse bone marrow cells. Adjuvanticity was evaluated in mice administered G9.1 with diphtheria toxoid (DT) through nasal vaccination.

**Results:**

G9.1 exhibited stronger IFN-α-inducing activity than A-class CpG ODN2216 and increased *T-bet/GATA-3* ratio by enhancing *T-bet* expression. Nasally administered G9.1 plus DT induced DT-specific mucosal IgA and serum IgG, but not IgE, responses with antitoxin activity in C57BL/6 and BALB/c mice, possibly due to IFN/BAFF production. Induction of T_H_1, but not T_H_2, -type Abs depended completely on pDCs, the first *in vivo* demonstration by CpG ODNs.

**Conclusions:**

G9.1 is a promising mucosal adjuvant for induction of pDC-mediated T_H_1 immunity.

## Introduction

Mucosal immunization has several distinct advantages over injection, including the stimulation of systemic immunity and mucosal immunity at the application site and other mucosa, including the lung and gastrointestinal tract mucosa [1]–[3]. However, the immunogenicity of synthetic proteins or peptide antigens is generally weak, whereas non-living vaccines administered at mucosal sites are possibly ineffective and can even lead to mucosal tolerance. Therefore, an adjuvant that potentiates the induction of appropriate immune responses to such antigens in the mucosa, as well as the organs, is required for the development of mucosal vaccines.

CpG oligodeoxynucleotides (ODNs), the synthetic counterparts of bacterial DNA, are currently tested in clinical trials as adjuvants for multiple immunotherapies, and these compounds have shown safety profiles similar to conventional vaccines [4]–[9]. The A class stimulates IFN-α production in plasmacytoid dendritic cells (pDCs), whereas the B class strongly activates B cells. Both activities are elicited upon binding to Toll-like receptor 9 (TLR9). The activation of pDCs and IFN-α production are important parameters in the assessment of influenza vaccine immunogenicity [10]. Certain vaccines, such as MMR (measles/mumps/rubella) and rabies vaccines, stimulate IFN-α production from pDCs in a manner similar to A class CpG ODNs [11]. On the other hand, the effects of CpG ODNs, primarily B class with a phosphorothioate (PS) backbone, were reported to differ with the administration route, schedule and sequence. In some cases, they may even cause lymphoid follicle destruction or immunosuppression in a pDC-independent manner [12]–[16]. In this regard, CpG ODNs with a phosphodiester (PO) backbone similar to bacterial DNA instead of PS, and capable of inducing IFN-α production, could be advantageous as adjuvants. They could induce T_H_1 immunity through activation of pDCs, and then processed inside the target cells.

Many studies have shown that palindromic (PO-type) CpG motifs are effective in inducing IFN-α production [17]–[20]. Based on our previous studies [17], [18], we recently developed a large series of PO-type CpG ODNs including G9.1 (GACGATCGTC linked in a unique manner by G9- and 1-mers at its 5′ and 3′ ends; Patent US7,718,623B2, May 2010). G9.1 exhibits stronger IFNα-inducing activity than A class CpG ODN2216, which is also composed of PO-GACGATCGTC, but linked to PO/PS-G 4- and 6-mers at its 5′ and 3′ ends to protect against nuclease degradation. In the present study, we investigated the adjuvanticity of G9.1 in an intranasal vaccination system using diphtheria toxoid (DT) as an antigen. DT mainly induces humoral immunity, but also T_H_2-mediated immunity when used with the well-known adjuvant cholera toxin [21], [22]. This combination allows further evaluation of T_H_1 immunity induction by G9.1. Protective immunity can be evaluated without the challenge experiment because international standards regarding antitoxin titer reflecting protection from diphtheria have been established [23]. Furthermore, DT is appropriate as an antigen for the investigation of mucosal immunity because *Corynebacterium diphtheriae* primarily infects the mucosal surface in the pharynx, larynx and nose. We demonstrated that G9.1 administration in an intranasal DT vaccination system raised DT-specific mucosal and serum Ab responses with a diphtheria-protective antitoxin activity. We also showed that pDCs were involved in the T_H_1-type Ab induction. Therefore, G9.1 appears to be a promising pDC-dependent PO-type T_H_1-enhancing CpG ODN for a future mucosal vaccine.

## Materials and Methods

### Oligodeoxynucleotides

All ODNs were synthesized by Hokkaido System Science Co., Ltd (Hokkaido, Japan). The sequences are shown in Fig. S1.

### Preparation and Culture of Human Cells

Peripheral blood mononuclear cells (PBMCs) and pDCs were isolated from the peripheral blood of healthy volunteers as described previously [19]. First, a low-density fraction of PBMCs was separated on 47.5% Percoll (GE Healthcare Bio-Sciences AB, Uppsala, Sweden). The pDC fraction was enriched as blood DC Ag (BDCA)4-positive cells by positive sorting with anti-BDCA4 (CD304)-Ab (Miltenyi Biotec Inc., CA, USA) and Dynabeads M-450 goat antimouse IgG (Invitrogen by Life Technology, Oslo, Norway). Alternatively, pDCs were enriched as lineage marker^−^/CD11c^−^/CD4^+^ cells by removing the cells reacting with Dynabeads CD14 (Invitrogen by Life Technology), followed by the anti-CD3/CD19/CD16/CD56/CD11c mAb (BD Biosciences Pharmingen, CA, USA) and Dynabeads M-450 goat anti-mouse IgG (Invitrogen by Life Technology). The positively sorted fraction contained >98% BDCA4^+^ cells, as assessed by counting the bead-binding cells. The lineage marker^−^/CD11c^−^/CD4^+^ fraction contained >85% pDC when analyzed by immunofluorescent staining with anti-CD304 or anti-BDCA-2 (CD303) (Miltenyi Biotec Inc.) Abs using flow cytometry (BD FACSCanto II; Becton, Dickinson and Company, NJ, USA). The cells were cultured in RPMI containing 2 mM l-glutamine, supplemented with 10% heat-inactivated fetal calf serum (FCS; Equitech-Bio, Ingram, TX; endotoxins <0.03 ng/mL), 100 U/mL penicillin, and 100 µg/mL streptomycin (37°C; 5% CO_2_). The use of human materials for research purposes was approved by the Ethics Committee of the Faculty of Medical Sciences, University of Fukui, Japan, and informed written consent was obtained from all participating subjects.

### Immunization of Mice and Sample Collection

Six-week-old BALB/c and C57BL/6 female mice were purchased from Japan SLC Co. (Shizuoka, Japan). TLR9 knockout (KO) mice were generously provided by Dr. Shizuo Akira (Osaka University, Osaka, Japan). All mice were maintained under pathogen-free conditions approved by the Institutional Animal Care and Use Committee of the National Institute of Infectious Diseases. On days 0, 14, 21, and 28, they were immunized intranasally under light ether anesthesia with 20 µL solution containing DT (courtesy of the Chemo-Sero-Therapeutic Research Institute, Kumamoto, Japan), DT plus G9.1, or DT plus recombinant cholera toxin B subunit (rCTB). They were sacrificed on day 35, unless stated otherwise in the Results section.

### Depletion of pDCs in Mice

To examine the dependence of immunogenicity on pDCs, 150 µg of anti-pDC Ab (anti-mPDCA-1 Ab: Miltenyi Biotec, GmbH, Bergisch Gladbach, Germany) or rat IgG2b (isotype control) was injected (i.v.) 24 h before and after each immunization. Depletion was confirmed 24 h after the injection by counting pDCs in splenocytes with FITC-conjugated anti-mPDCA-1 Ab using the JSAN™ cell sorting and analysis system (Bay Bioscience Co., Ltd., Kobe, Japan).

### Measurement of DT-specific Ab and Diphtheria Antitoxin Titers

The DT-specific IgG, IgA, and IgE Ab titers were determined by ELISA [24]. The Ab titers were expressed as the highest endpoint dilution of each sample providing a positive reaction, and the Ab titers were analyzed on a logarithmic scale.

Titration of the mouse serum diphtheria antitoxin was performed as described by Miyamura et al. [23], and the titers were expressed in international units (IU)/mL.

### Measurement of Cytokine Concentrations

Cytokine concentrations in culture supernatants were measured using ELISA kits (Biosource International Inc. or Invitrogen Co., Camarillo, CA), Quantikine immunoassay (R&D Systems Inc., Minneapolis, MN), Verikine™ Human IFN-α Multi-subtype ELISA Kit (PBL InterferonSource, Piscataway, NJ), Milliplex MAP Kit (Millipore Co., Billerica, MA), or Invitrogen’s Multiplex Bead Immunoassay kits according to the manufacturer’s instructions.

### Real-time RT-PCR

Total RNA was extracted using ISOGEN (Nippon Gene, Toyama, Japan) and converted to cDNA using a SuperScript first-strand synthesis system for RT-PCR (Invitrogen Life Technologies, Carlsbad, CA) or a PrimeScript RT reagent Kit (Takara Bio Inc., Otsu, Japan). For real-time PCR of human *T-bet*, *GATA-3*, and *B2M*, cDNA was analyzed in an MJ Mini™ Personal Thermal Cycler (Bio-Rad, Hercules, CA) using iQ Supermix (Bio-Rad) with TaqMan Gene Expression Assays (Applied Biosystems, Carlsbad, CA). For the expression of mouse genes, cDNAs were analyzed in an ABI Prism 7900 Sequence Detection System (Applied Biosystems) using SYBR Green PCR Master Mix (Applied Biosystems). The mouse PCR primer sequences are shown in Fig. S1.

### Histopathology and Immunological Parameters

The brain, pharynx, lung, liver, kidney, spleen, intestine, and skin of mice were fixed with formalin and stained with H&E. Immunological parameters of lung and nasal cavity lavages, feces, and blood were tested as described previously [24].

### Statistical Analysis

The data were expressed as mean and standard error (SE). Multiple group comparisons were analyzed by ANOVA with post-hoc Tukey’s tests for pair-wise comparisons. Two-group analyses were performed by paired or unpaired *t*-tests as indicated. All *p* values of <0.05 were considered statistically significant.

## Results

### G9.1 Enhanced T_H_1 Immunity in Human Peripheral Blood Mononuclear Cells

**Figure 1:**
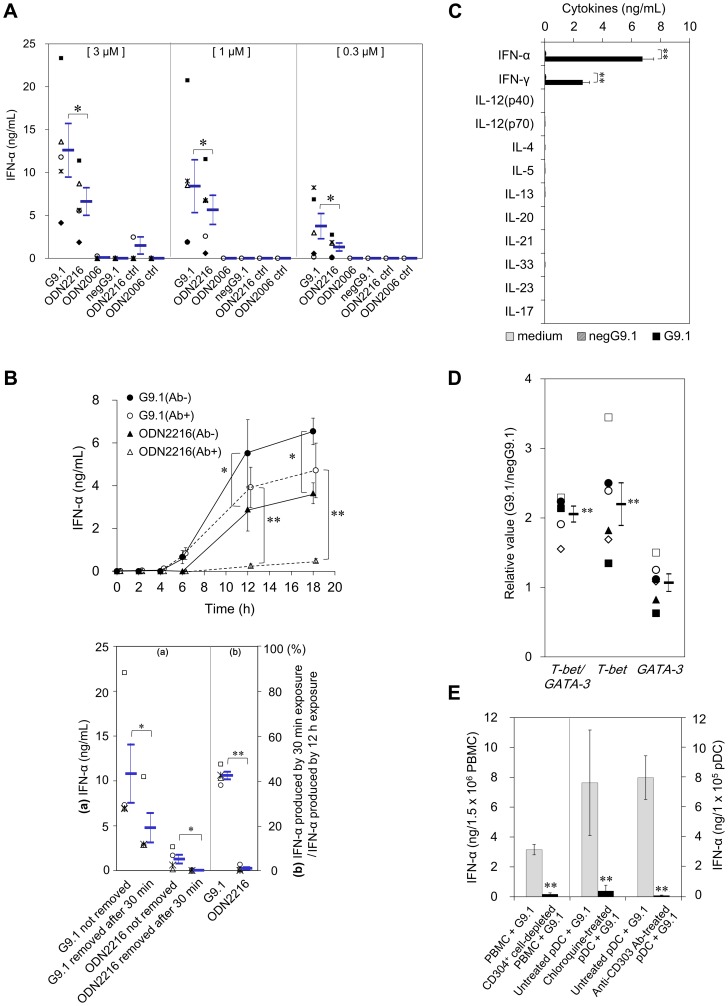
G9.1 stimulated T_H_1-related cytokine production and transcription factor expression in human PBMCs. ***A***, PBMCs (1.5×10^6^/mL) were cultured for 16 h with various concentrations of CpG ODNs or their negative controls. The IFN-α concentration released in the culture supernatant was measured by ELISA. (five donors; each symbol reflects an individual donor; *p<0.05 between G9.1 and ODN2216). ***B***, PBMCs (1.5×10^6^/mL) were cultured for 2, 4, 6, 12, or 18 h with 1 µM G9.1 or ODN2216 in the presence/absence of anti-human IFN-α/β receptor-chain 2 Ab (4 µg/mL; PBL Interferon Source, NJ, USA) (upper panel, six donors); or treated with IFN-α/β receptor-chain 2 Ab (30 min), exposed to 1 µM G9.1 or ODN2216 (30 min), and re-cultured for 12 h after removing/not removing the CpG ODNs by extensive washing (lower panel, four donors). The addition of 4 µg/mL mouse IgG2a as isotype control did not alter G9.1-mediated IFN-α production. *p<0.05 and ** *p*<0.01 by a paired *t*-test. ***C and D***, PBMCs (1.5×10^6^/mL) were cultured for 12–16 h (C) or overnight (D) in medium alone, 0.4 µM negG9.1 or 0.4 µM G9.1, cytokine concentrations in the culture supernatant measured (C), and RT-PCR performed for the cell pellet to estimate *T-bet* and *GATA-3* expression levels (D). **p<0.01 by a paired *t*-test (six donors). ***E***, PBMCs, CD304^+^ cell-depleted PBMCs, and pDCs untreated or pretreated with 1 µg/mL chloroquine or 2 µg/mL anti-CD303 Ab for 30 min were cultured for 12–16 h with 0.4 µM G9.1 and IFN-α concentrations in the supernatant compared with native G9.1-treated cultures. Data shown are three donors for PBMC, six donors for choloroquine-treated assay and seven donors for anti-CD303. **p<0.01 by a paired *t*-test. In all the experiments, the IFN-α concentrations in the media from PBMCs or pDCs treated with negG9.1 or left untreated were negligible.

We previously demonstrated that CpG ODNs longer than 18-mer are required to stimulate IFN-α production [25]. Here we determined the optimal length and position of the PO-type poly(G) linked to PO-GACGATCGTC [18] for efficient IFN-α production by human PBMCs. The G9.1 (GGGGGGGGGGACGATCGTCG) was the most effective sequence, exhibiting stronger activity than the prototype A-class CpG, ODN2216 (Fig. 1A). Induction of IFN-α production by G9.1 was dependent on the sequence GACGATCGTC. The substitution of CG for GC, or the substitution of AT for TA, AA, or TT, removed this effect (data not shown).

The involvement of IFN-αβ receptors in ODN-mediated PBMC responses was determined by time-course analysis. We demonstrated that G9.1 induced IFN-α production in the presence of anti-human IFN-αβ receptor-chain 2 Ab (Fig. 1B, upper panel). We confirmed that this antibody blocks IFN-α responses from pDCs in our preliminary experiments. The IFN-αβ receptor-independent IFN-α production accounted for 79.3% ±16.3% of the total after 12 h of incubation with G9.1. In contrast, this independent response was only 12.6% ±3.3% of the total in the presence of ODN 2216, which was previously reported to induce IFN-α by a mechanism dependent on IFN-αβ receptors [26]. When G9.1 was removed after a 30-min exposure, the IFN-α production was 42.3% ±1.9% of the total amount produced by continuous exposure for 12 h, compared with 0.9% ±0.6% for ODN2216 (Fig. 1B, lower panel). Therefore, G9.1 generates a stronger IFN-αβ receptor-independent IFN-α production than ODN2216.

Incubation of PBMCs with G9.1, but not control ODN for G9.1 (negG9.1), also upregulated IFN-γ production (Fig. 1C). No other T_H_1/T_H_2/T_H_17-related cytokines were observed in PBMC culture supernatants. Consistent with the cytokine production pattern, expression of *T-bet*, a transcriptional regulator essential for T_H_1 cell differentiation, increased more than expression of *GATA-3,* a transcription factor required for T_H_2 cell differentiation, resulting in an increase in the *T-bet*/*GATA-3* ratio (Fig. 1D).

Depletion of pDCs (CD304-positive cells) in PBMCs abrogated G9.1-induced IFN-α production. In addition, treatment of PBMCs with an inhibitor of endosomal maturation (chloroquine) or CD303 Ab inhibited IFN-α production (Fig. 1E). These results imply that G9.1-induced IFN-α production depends on pDCs expressing endosomal TLR9.

### G9.1 Upregulated T_H_1 Immunity in Mice

**Figure 2:**
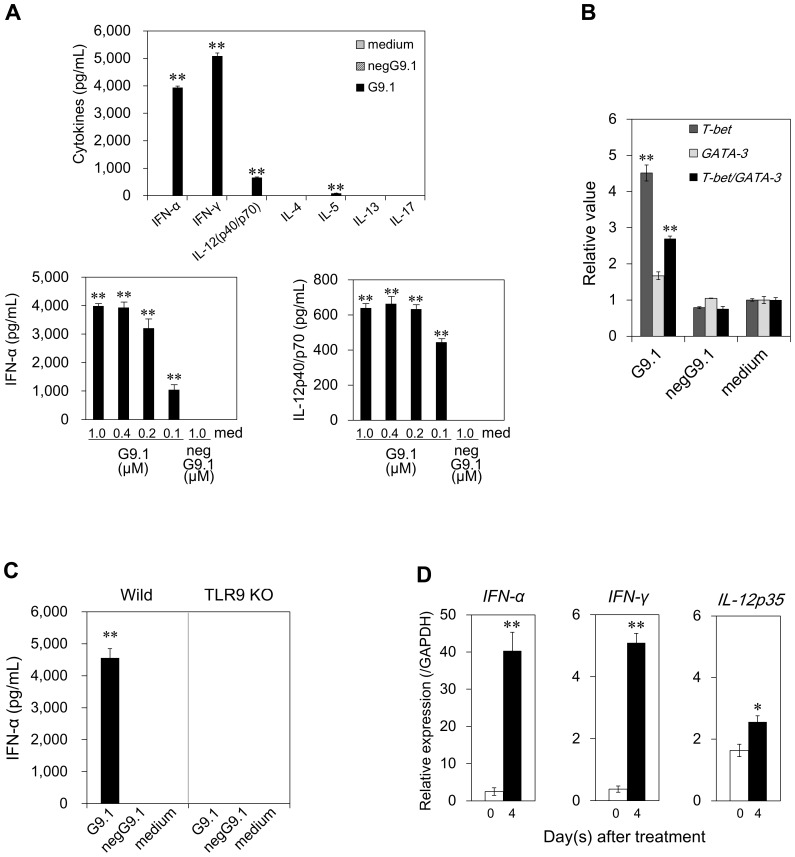
G9.1 upregulated T_H_1-related cytokine production and transcription factor expression in mice. ***A and B,*** BM cells (5×10^6^/mL) prepared from C57BL/6 mice were cultured for 24 h in the medium alone, 0.4 µM G9.1, 0.4 µM negG9.1 (A, upper; B), or with varying concentrations of G9.1 or negG9.1 (A, lower). Cytokine production was measured by ELISA and *T-bet* and *GATA-3* expression levels were assessed by RT-PCR. Data shown are from one of the two experiments that produced almost identical results (n = 2; **p<0.01 compared with medium alone). ***C***, BM cells isolated from wild-type C57BL/6 (Wild) and TLR9 KO mice were cultured in the presence of G9.1 (0.4 µM), negG9.1 (0.4 µM), or medium. IFN-α level in culture supernatant was assessed. **p<0.01 compared with medium control (a *t*-test, n = 2). ***D***, Mice were injected (i.p.) with 50 µg of G9.1 or PBS. After 4 days, splenocytes were assessed for the expression of *IFN-α*, *IFN-γ*, and *IL-12p35* by RT-PCR. Data are relative expression levels (n = 4; *p<0.05 and **p<0.01 compared with respective controls; paired *t*-test).

In mouse bone marrow (BM) cells, G9.1, but not negG9.1, induced cytokine production (typically represented by IFN-α and IFN-γ), resembling the profile in human PBMCs except for differences in IL-12 and (to a lesser extent) IL-5 expression levels (Fig. 2A). Consistent with the T_H_1 cytokine expression profile, the ratio of *T-bet*/*GATA-3* expression increased (Fig. 2B). BM cells prepared from TLR9 KO mice did not respond to G9.1, as shown by the undetectable level of IFN-α in their culture supernatants (Fig. 2C).

The *in vivo* effect was then assessed by examining the expression levels of *IFN-α*, *IFN-γ*, and *IL-12p35* in splenocytes 4 days after of injection (i.p.) with G9.1 or PBS. G9.1 produced a significant increase in IFN-α and IFN-γ expression (Fig. 2D). The upregulation of T_H_1 immunity by G9.1 in both humans and mice prompted us to investigate G9.1 as an adjuvant for a mucosal vaccine in mice.

### Nasally Administered G9.1 Exhibited Phylactic Adjuvanticity through TLR9

**Figure 3:**
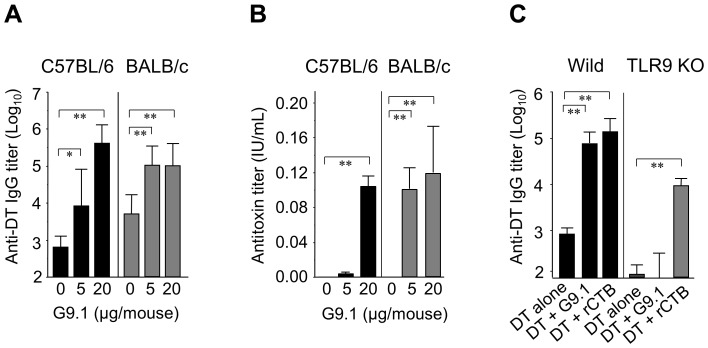
In the nasal vaccination system, G9.1 enhanced DT-specific TLR9-dependent IgG response and antitoxin activity. ***A and B***, C57BL/6 and BALB/c mice were administered DT (2 Lf/mouse) plus G9.1 (0, 5, or 20 µg/mouse) nasally on days 0, 14, 21, and 28. Serum anti-DT IgG (A) and antitoxin (B) titers were measured in the blood samples collected from the tail on day 35. ***C***, Wild-type C57BL/6 (Wild) and TLR9 KO mice were immunized with DT (2 Lf) alone, DT (2 Lf) plus G9.1 (20 µg), or DT (2 Lf) plus rCTB (10 µg), and serum anti-DT IgG titers measured as in ***A***. *p<0.05 and **p<0.01 as determined by ANOVA followed by post hoc Tukey’s test (n = 5). Results were nearly identical in three independent experiments.

Each mouse (C57BL/6 and BALB/c) was administered 50 µg of G9.1 nasally with or without 2 Lf of DT on days 0, 14, 21, and 28. No histological changes were observed in the brain, pharynx, lung, liver, kidney, spleen, or intestine as revealed by formalin-fixed tissue samples harvested on day 42 and stained with H&E (data not shown). Other mice were nasally administered 2 Lf of DT alone, DT plus 5 or 20 µg of G9.1, or DT plus 10 µg of rCTB (a well-known adjuvant) on days 0, 14, 21, and 28. Peripheral blood samples were withdrawn on day 35 and serum titers of DT-specific Abs were measured. When DT alone was administered, the serum titers of anti-DT IgG Ab were as low as approximately 10^3^ and 10^4^ in C57BL/6 and BALB/c mice, respectively. In contrast, when G9.1 was co-administrated, the titers increased 10–1,000-fold, reaching maximal levels at 20 µg of G9.1 in C57BL/6 and 5 µg of G9.1 in BALB/c mice (Fig. 3A). Sera isolated after treatment with 2 Lf DT plus 20 µg of G9.1 (C57BL/6) or 2 Lf DT plus 5 µg of G9.1 (BALB/c) exhibited protective antitoxin activity, whereas the sera of mice that received DT alone did not (Fig. 3B). Anti-DT IgA titer increased in sera from mice co-administered G9.1 (Fig. S2); however, anti-DT IgE was not detected (data not shown).

In contrast to wild-type C57BL/6 mice, an increase in the anti-DT IgG response by G9.1 was not observed in TLR9 KO mice immunized using the same protocol (Fig. 3C), indicating that G9.1 adjuvanticity is mediated by TLR9. Although the response to DT alone was also lower in TLR9 KO mice, the interpretation of the results is not affected because the TLR9 KO mice produced IgG in response to rCTB, an adjuvant not dependent on TLR9.

### Nasally Administered G9.1 Stimulated Plasmacytoid Dendritic Cell-mediated T_H_1-type Ab Production

**Figure 4:**
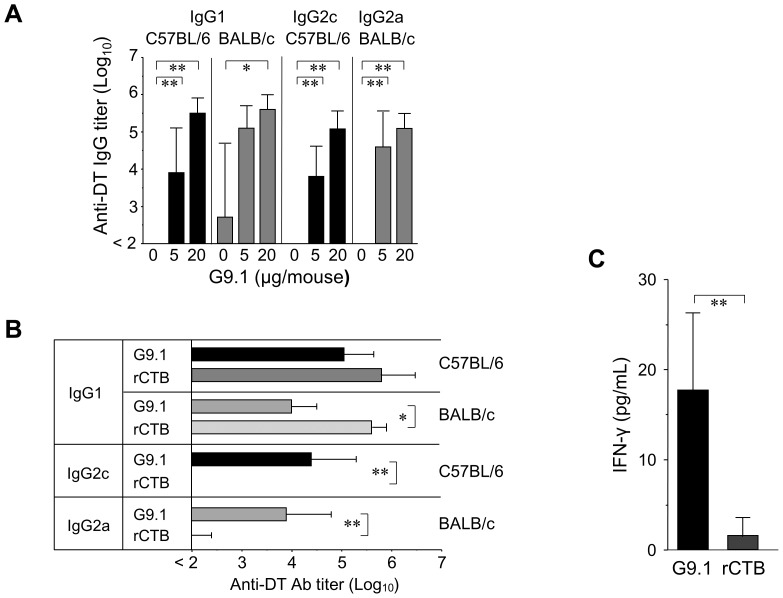
Nasally administered G9.1 induced both IgG1 and IgG2a/c production. ***A***, C57BL/6 and BALB/c mice were immunized as described in Fig. 3. ***B,*** C57BL/6 and BALB/c mice were administered DT (2 Lf) with G9.1 (20 µg) or rCTB (10 µg). Serum titers of anti-DT IgG1, IgG2a (BALB/c), and IgG2c (C57BL/6) Abs were measured as in Fig. 3. *p<0.05 and **p<0.01 as determined by ANOVA followed by post hoc Tukey’s test or by *t*-test; n = 5. Experiment A and B were repeated thrice and twice, respectively, yielding nearly identical results. ***C,*** Splenocytes isolated from BALB/c mice vaccinated with DT (2 Lf) plus G9.1 (20 µg) or DT plus rCTB (10 µg) were cultured for 24 h with 10 µg/mL DT. IFN-γ concentration in the culture supernatant was measured by ELISA. **p<0.01 as determined by a *t*-test (n = 3).

Co-administration of G9.1 and DT enhanced the production of IgG2a (BALB/c) and IgG2c (C57BL/6) as well as IgG1 (Fig. 4A). To confirm the advantages of G9.1 as a vaccine adjuvant, we compared the serum titers of these Abs after DT and G9.1 treatment with the serum titers after combined DT and rCTB treatment. As shown in Fig. 4B, G9.1 administration elicited the production of IgG1, IgG2a, and IgG2c subclasses, whereas rCTB treatment induced only IgG1 production both in C57BL/6 and BALB/c mice. These data suggest that G9.1 exerts its adjuvanticity by enhancing T_H_1 immunity in a manner different from rCTB. This was supported by the induction of IFN-γ in splenocytes prepared from mice vaccinated with DT plus G9.1, but not from mice vaccinated with DT plus rCTB (Fig. 4C).

**Figure 5:**
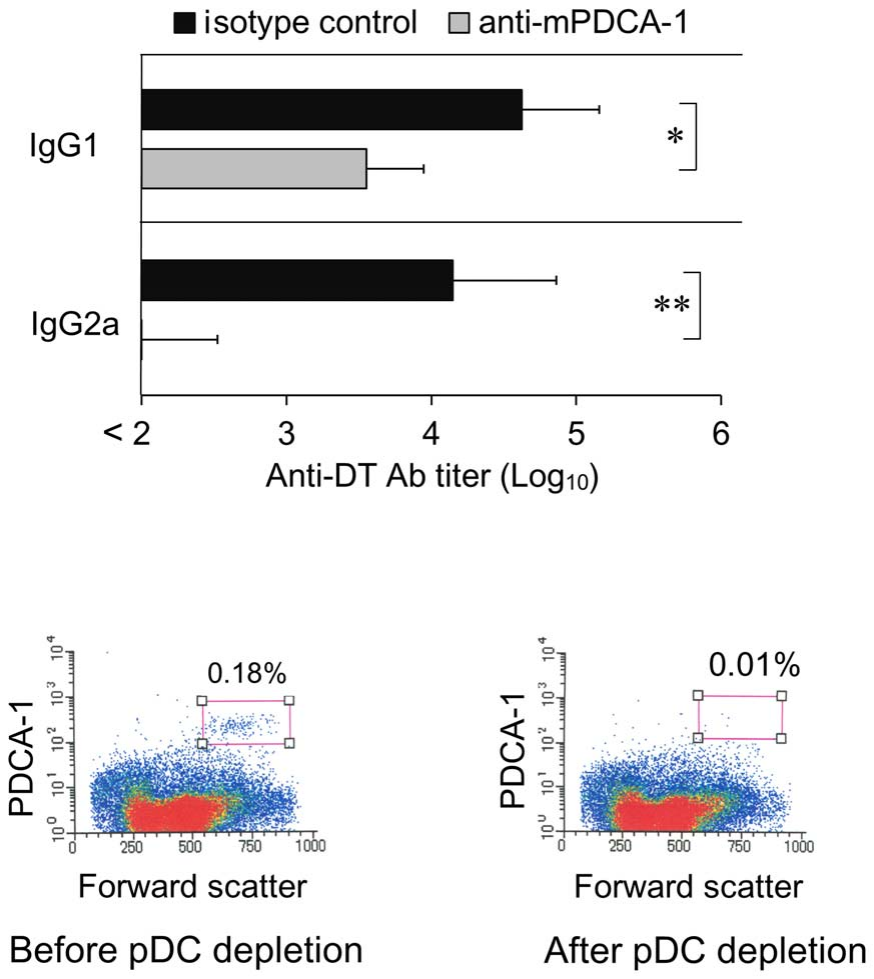
G9.1-induced T_H_1-type Ab production was completely pDC-dependent. Anti-mPDCA-1-treated and isotype control-treated BALB/c mice (n = 4) were administered DT (2 Lf) plus G9.1 (20 µg) nasally twice at a 1-month interval. Serum titers of anti-DT IgG1 and IgG2a were measured as described in Fig. 3. The percentage of pDCs in splenocytes decreased from 0.18 to 0.01 after anti-mPDCA-1 treatment (lower panel). The pDC-depleted mice exhibited no G9.1-induced IgG2a production (upper panel). *p<0.05 and **p<0.01 as determined by *t*-test. Three independent experiments yielded nearly identical results.

We then investigated the involvement of pDCs in the induction of T_H_1-type Ab production. BALB/c mice depleted of pDCs by mPDCA-1 Ab treatment and control mice treated with rat IgG2b were immunized with 2 Lf DT plus 20 µg of G9.1 twice at a 1-month interval, and anti-DT IgG2a and IgG1 titers measured in blood withdrawn one month after the last immunization. In anti-mPDCA-1-treated mice, the IgG2a titer did not increase with G9.1 administration and the IgG1 titer was 10 times lower than that in control mice (Fig. 5). These results suggest that nasally administered G9.1 specifically stimulates pDC-mediated T_H_1 immunity.

### Nasally Administered G9.1 Enhanced Diphtheria Toxoid-specific Mucosal IgA Production

**Figure 6:**
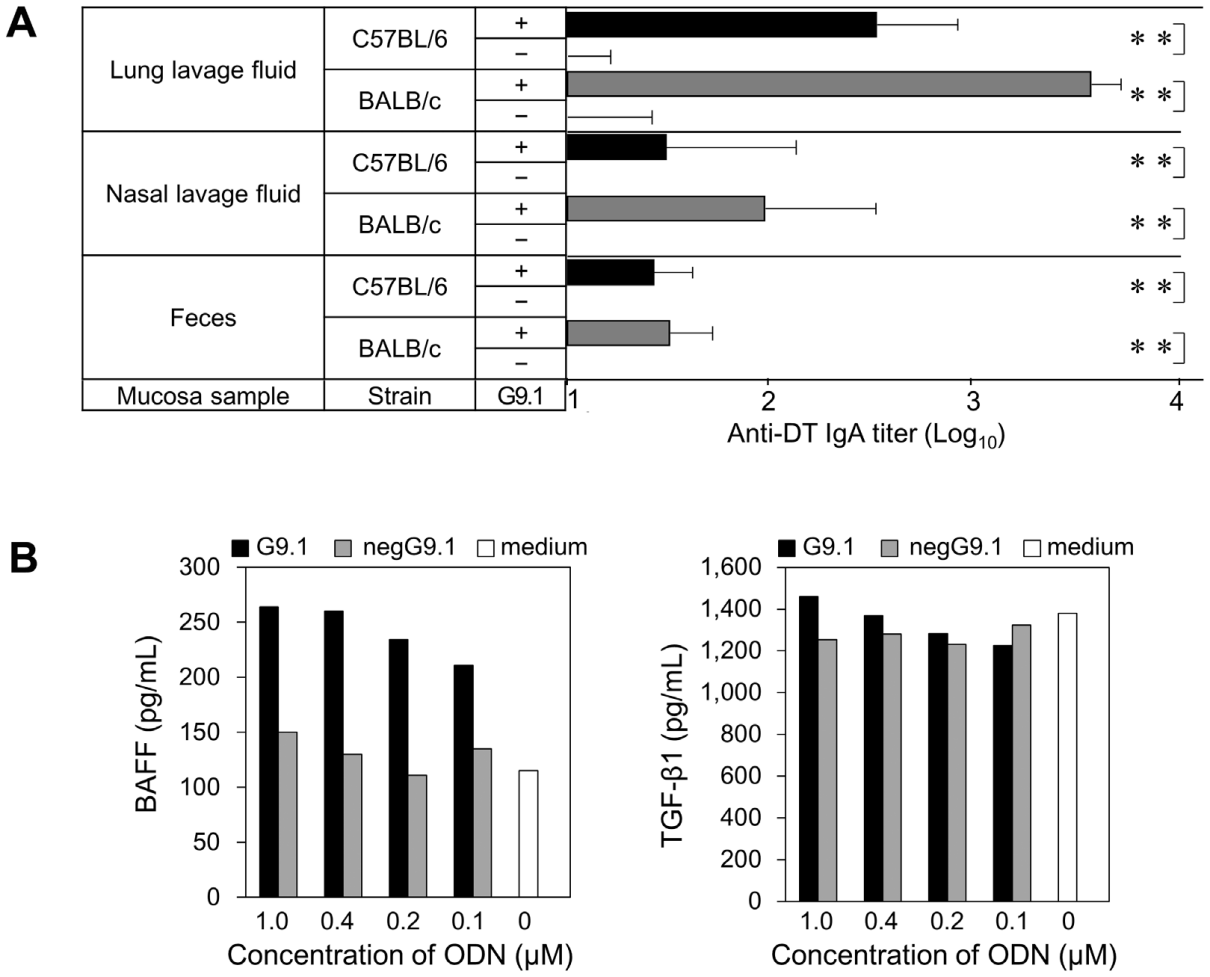
Nasally administered G9.1 promoted mucosal IgA response. ***A***, BALB/c and C57BL/6 mice were immunized intranasally with 2 Lf DT with or without 20 µg of G9.1 on days 0, 14, 21, and 28. Lavages from the lungs and nasal cavity and the supernatants of the feces mixture were prepared on day 35 and anti-DT IgA titers measured. **p<0.01 by *t*-test (n = 5). Three independent experiments yielded nearly identical results. ***B***, BM cells (5×10^6^/mL) prepared from C57BL/6 mice were cultured for 24 h in medium alone or with varying concentrations of G9.1 or negG9.1. The culture supernatants were evaluated for BAFF and the activated form of TGF-β1 by Quantikine immunoassay (ELISA Kit). Data are from one of two independent experiments yielding nearly identical results. Shown as the means from duplicate assays.

The development of humoral immunity through the secretory form of IgA would be preferred for the prevention of microbial invasion. We measured DT-specific IgA titers in lung and nasal cavity lavages and feces to assess the response in the lumen of the lung, nasal mucosa, and intestinal mucosa to nasal administration of 2 Lf of DT alone or 2 Lf of DT plus 20 µg of G9.1. Nasal administration of G9.1 enhanced IgA production not only at the mucosal sites near the induction site, such as the nasal cavity and lungs, but also at the distant sites, such as intestines (Fig. 6A).

To examine the mechanism for the induction of IgA production, the production of TGF-β_1_ and BAFF, which stimulate IgA class switching, was evaluated in mouse BM cells. G9.1-stimulated BM cells produced higher BAFF than unstimulated BM cells (Fig. 6B), suggesting that G9.1-enhanced BAFF synthesis may contribute to IgA production.

## Discussion

We demonstrate the mucosal and systemic adjuvanticity of a uniquely designed completely PO-bond CpG ODN, G9.1, which induces a pDC-mediated T_H_1-type immune response.

As a potent adjuvant, G9.1 appears to have several unique advantages. Due to the PO-backbone, it is predicted to behave identical to natural bacterial DNA, triggering immunity before being degraded by host nucleases. Induction of IFN-α production was higher than that of a well-known A class CpG, ODN2216, in humans (Fig. 1A), and observed even in mice (Fig. 2A), which enables evaluation of the adjuvanticity. The substantial induction in the early phase of the culture (Fig. 1B) supports its potential for vaccine development. Moreover, the pDC-mediated induction of T_H_1 immunity by nasal administration may support the appropriateness of G9.1 as a mucosal adjuvant because the well-known adjuvant rCTB did not induce T_H_1 immunity (Fig. 4B/C).

There are several classes of immunostimulatory CpG ODNs: B, A, C, and P. G9.1 resembles an A class CpG in that it contains one palindromic CpG motif and induces the production of large quantities of IFN-α through pDC TLR9. However, unlike ODN2216, the G9.1-mediated IFN-α production was largely independent of the type I IFN receptor. This may be explained by the unique conformation conferred by asymmetric PO-Gs and palindromic CpG motif. We previously reported that NF-κB activation and *de novo* expression of *IRF-7* in human pDC involved a type I IFN receptor-independent mechanism, which was induced by the former PO-type CpG-ODN G10 (GACGATCGTC with 10 mer Gs at both ends; weaker activity than G9.1) [19], [27]. The precise mechanism of G9.1 effects are under investigation.

In our vaccination system, G9.1-induced T_H_1-related Ab production was dependent on pDCs (Fig. 5), the first such report *in vivo* and consistent with our *in vitro* results showing the involvement of pDCs in G9.1-induced IFN-α production (Fig. 1E). Even under conditions where T_H_2 immunity should have been preferentially induced, as by DT administration [21], substantial amounts of IgG2a/c Ab were produced by G9.1 administration (Fig. 4). Such switching from T_H_2 to T_H_1 immunity has been reported in other studies but only for B class PS-CpG [28], [29]. In this sense, G9.1 could be defined as a pDC-dependent PO-type T_H_1-enhancing CpG ODN because its structural characteristics are distinct from other CpG ODNs and its adjuvanticity was demonstrated to be pDC-dependent.

It has been reported that mucosal infections increase the number of pDCs [30], [31] and that nasal administration of CpG ODNs in mice results in selective recruitment of pDCs into the lung [32]. On the basis of these reports and our data, we speculate that pDCs are recruited and activated in the mucosa of the respiratory system following nasal administration of G9.1. This process, resulting in the production of cytokines (e.g., IFN-α and IFN-γ) may constitute the central mechanism in the development of the T_H_1-polarized immune response as evidenced by an increase in the ratio of *T-bet/GATA-3* expression (Fig. 2B), IgG2a/c Ab production (Fig. 4A/B), and IFN-γ production (Fig. 4C).

The production of IgG2a/c by G9.1 may result from IFN-α and IFN-γ production (Figs. 2A/D and 4C) because both type I and type II IFN have been shown to stimulate the production of these IgG subclasses [33]–[37]. In the DT vaccination system, G9.1 also triggered IgG1 Ab production (Fig. 4A/B). This may be due to concomitant production of IL-12 and IFN-γ [38] because the production of these two proteins, but not of IL-4, was increased by G9.1 (Fig. 2A). However, IgG1 production may not be solely due to G9.1-activated pDCs because G9.1-induced IgG1 production was still observed in pDC-depleted mice (Fig. 5), suggesting the involvement of other TLR9-expressing cells.

The principal advantage of mucosal vaccines is that antigens can be neutralized before systemic invasion. Although antitoxin activity was detected in the sera of G9.1-injected mice (Fig. 3B), we could not determine antitoxin activity directly in mucosal preparations owing to dilution of secretory fluid by the washing solution (also cytotoxic). Nonetheless, we provide evidence that G9.1 also induces DT-specific IgA secretions from mucous membranes of aerodigestive tracts (Fig. 6A).

It is unclear how G9.1 enhances mucosal IgA production. One possibility is increased epithelial transport of IgA by IFN-γ-mediated upregulation of the polymeric immunoglobulin receptor (PIGR) because IFN-γ is known to upregulate *PIGR* [39]. It has also been demonstrated that the switching of uncommitted IgM^+^ B cells to IgA-expressing cells is directed by TGF-β_1_ and CD40L [35], [40]. Recently, Tezuka et al. (2011) reported that pDCs in gut-associated lymphatic tissue play a critical role in T cell-independent IgA production by expressing APRIL and BAFF, the TNF family ligands inducing IgA production [41]. Our results also suggest that G9.1-induced BAFF production (Fig. 6B) may contribute to upregulation of IgA production in the nasal DT-vaccination system. No alteration in the level of TGF-β even by the culture with G9.1 may be ascribed to its constitutive production. The cells responsible for BAFF production are currently under investigation.

Many vaccines cause allergic reactions in susceptible individuals, and use of CpG ODNs is a promising strategy to circumvent allergic responses. pDCs appear to suppress allergic responses through enhancement of T_H_1 immunity. G9.1 increased *T-bet* expression but did not decrease *GATA-3* expression (Figs. 1D and 2B). However, the G9.1-mediated increase in IgG responses may reduce IgE responses, leading to suppression of allergic inflammation. Thus, vaccination with G9.1 may be particularly advantageous, not only to induce phylaxis, but also to control ongoing inflammation. The data supporting this notion are presented in the annex (Fig. S3).

Most protein antigens exhibit poor immunogenicity when administered mucosally and can even induce immunological tolerance. In addition, antigens administered mucosally must survive degradation by luminal enzymes and trapping by mucus. Therefore, much effort is currently being devoted to the development of an effective adjuvant that triggers protective immunity to combat infectious microbes at the mucosal surface. Given the demonstrated phylactic, T_H_1-inducing, and anti-allergic effects shown here, we propose G9.1 as a promising mucosal adjuvant for the development of novel vaccines, such as oral and nasal vaccines, to overcome emerging and re-emerging infectious diseases. The mechanisms for G9.1 adjuvanticity and optimal methods for mucosal vaccination warrant intensive study.

## Supporting Information

Figure S1 **Sequences of ODNs and primers of mouse genes used in this study.** Upper table shows the sequences of CpG ODNs and their negative controls. Bases in capital letters are phosphodiester and those in lower case phosphorothioate. Lower table shows the primer sequences for RT-PCR of mouse genes.
(TIF)

Figure S2 **Nasally administered G9.1 raised DT-specific IgA response in sera.** C57BL/6 and BALB/c mice were nasally vaccinated and serum titers of anti-DT IgA measured according to the protocol described in Fig. 3. *p<0.05 and **p<0.01 as determined by ANOVA, followed by post hoc Tukey’s test (n = 5).
(TIF)

Figure S3 **G9.1 suppressed ovalbumin-induced allergic reaction.** Male BALB/c mice received an i.p. injection of 10 µg of ovalbumin (OVA) in alum on days 0 and 21 and were challenged on day 35 with an i.c. injection of PBS, 5 µg of OVA, or 5 µg of OVA plus 50 µg of G9.1 (into the ear). One day later, ear thickness was measured and histological and immunological parameters at the injection site were analyzed. Ear thickness increased 1.043±0.024-fold (mean ± SD, n = 4, *p*<0.05, paired t-test) in OVA-challenged mice. But no increase was observed (0.985±0.019, n = 5, NS) when G9.1 was injected with OVA. Injection of PBS alone did not cause ear thickening. A marked infiltration of leukocytes including lymphocytes, eosinophils, and neutrophils was observed in the dermis and hypodermis of the OVA-challenged mice. Immunocyte infiltration was substantially reduced by G9.1 injection (***A***). The OVA challenge increased *GATA-3* mRNA expression, but not *T-bet* mRNA expression. When G9.1 was co-injected, *T-bet* expression increased markedly without significant change in *GATA-3* expression, thus resulting in an increased *T-bet/GATA-3* ratio (***B***). *p<0.05 as determined by ANOVA followed by post hoc Tukey’s test (n = 5 for PBS, n = 4 for OVA, and n = 5 for OVA plus G9.1).
(TIF)
