# Expression of Concern: A Palindromic CpG-Containing Phosphodiester Oligodeoxynucleotide as a Mucosal Adjuvant Stimulates Plasmacytoid Dendritic Cell-Mediated T_H_1 Immunity

**DOI:** 10.1371/journal.pone.0279154

**Published:** 2023-01-11

**Authors:** The PLOS ONE Editors

The Funding Statement for this article [1] states that the study received funding from the Smoking Research Foundation, which according to [2] has received financial support from the tobacco industry. In light of this issue the *PLOS ONE* article [1] does not comply with the journal’s policy on Funding from Tobacco Companies [3] which was implemented in 2010. Therefore, the *PLOS ONE* Editors issue this Expression of Concern.

We regret that this concern was not identified and addressed prior to the article’s [1] publication.
