# Supplementary figures and images for: A Palindromic CpG-Containing Phosphodiester Oligodeoxynucleotide as a Mucosal Adjuvant Stimulates Plasmacytoid Dendritic Cell-Mediated TH1 Immunity
Source: PLoS One. 2014 Feb 24;9(2):e88846. doi: 10.1371/journal.pone.0088846 (PMC3933336; doi:10.1371/journal.pone.0088846)

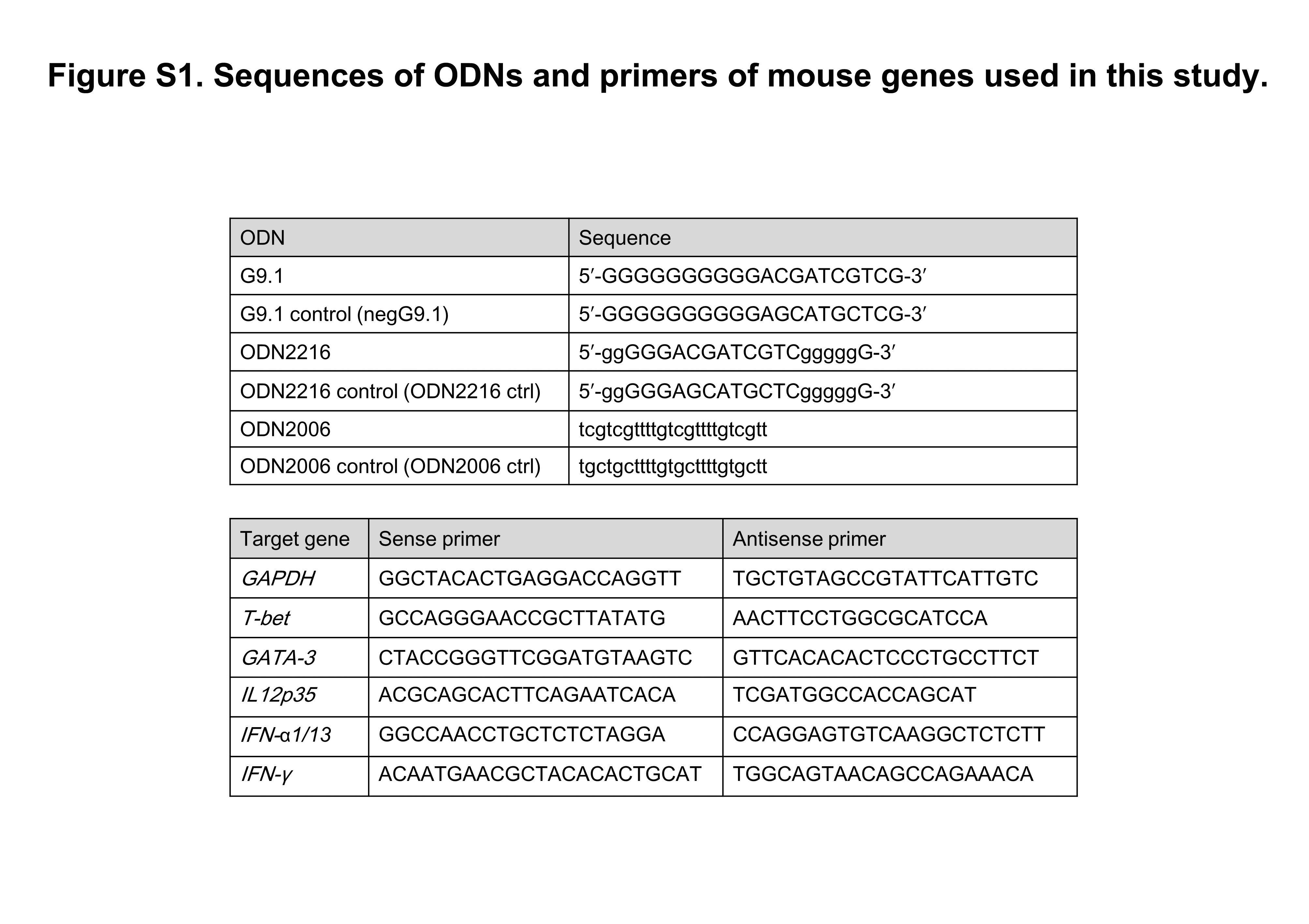

Supplement: Figure S1 — Sequences of ODNs and primers of mouse genes used in this study. Upper table shows the sequences of CpG ODNs and their negative controls. Bases in capital letters are phosphodiester and those in lower case phosphorothioate. Lower table shows the primer sequences for RT-PCR of mouse genes. (TIF) [file pone.0088846.s001.tif]

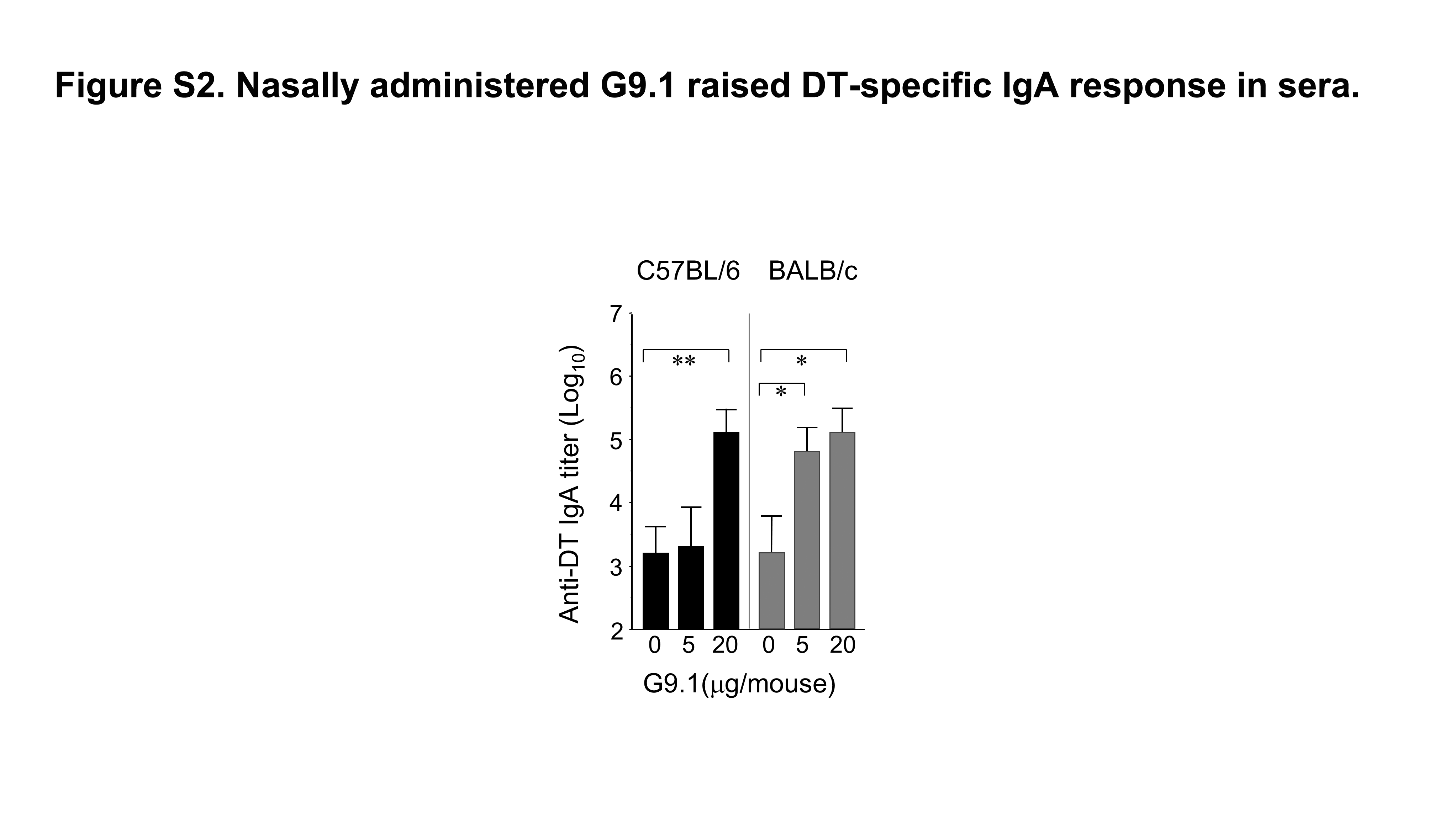

Supplement: Figure S2 — Nasally administered G9.1 raised DT-specific IgA response in sera. C57BL/6 and BALB/c mice were nasally vaccinated and serum titers of anti-DT IgA measured according to the protocol described in Fig. 3. *p<0.05 and **p<0.01 as determined by ANOVA, followed by post hoc Tukey’s test (n = 5). (TIF) [file pone.0088846.s002.tif]

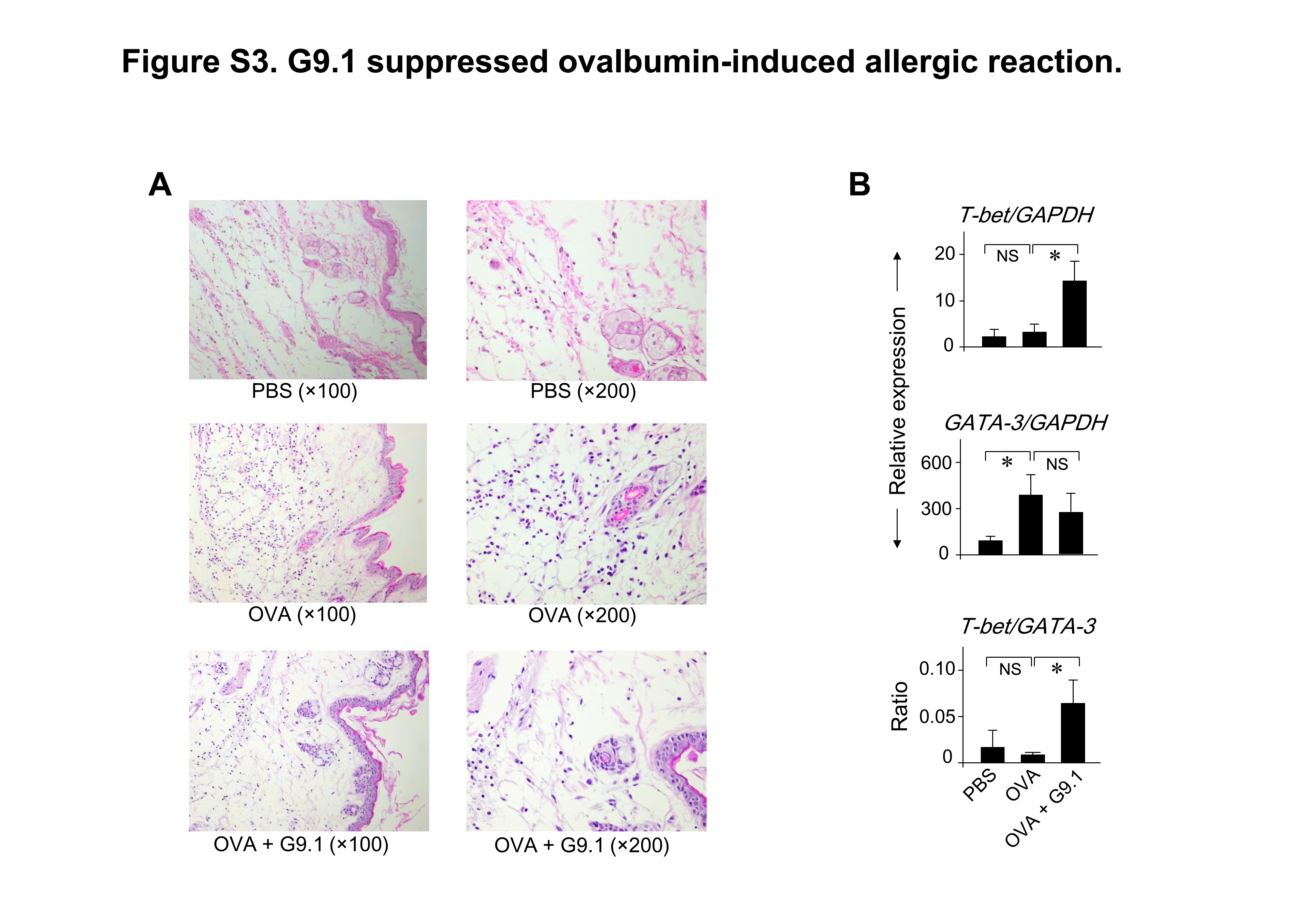

Supplement: Figure S3 — G9.1 suppressed ovalbumin-induced allergic reaction. Male BALB/c mice received an i.p. injection of 10 µg of ovalbumin (OVA) in alum on days 0 and 21 and were challenged on day 35 with an i.c. injection of PBS, 5 µg of OVA, or 5 µg of OVA plus 50 µg of G9.1 (into the ear). One day later, ear thickness was measured and histological and immunological parameters at the injection site were analyzed. Ear thickness increased 1.043±0.024-fold (mean ± SD, n = 4, p<0.05, paired t-test) in OVA-challenged mice. But no increase was observed (0.985±0.019, n = 5, NS) when G9.1 was injected with OVA. Injection of PBS alone did not cause ear thickening. A marked infiltration of leukocytes including lymphocytes, eosinophils, and neutrophils was observed in the dermis and hypodermis of the OVA-challenged mice. Immunocyte infiltration was substantially reduced by G9.1 injection (A). The OVA challenge increased GATA-3 mRNA expression, but not T-bet mRNA expression. When G9.1 was co-injected, T-bet expression increased markedly without significant change in GATA-3 expression, thus resulting in an increased T-bet/GATA-3 ratio (B). *p<0.05 as determined by ANOVA followed by post hoc Tukey’s test (n = 5 for PBS, n = 4 for OVA, and n = 5 for OVA plus G9.1). (TIF) [file pone.0088846.s003.tif]
